# Determination of a cutoff value for pelvic floor distensibility using the Epi-no balloon to predict perineal integrity in vaginal delivery: ROC curve analysis. Prospective observational single cohort study: Determinação de um valor de ponto de corte para a extensibilidade do assoalho pélvico pelo balão Epi-no para predizer integridade perineal no parto vaginal: análise pela curva ROC. Estudo prospectivo observacional de coorte única

**DOI:** 10.1590/1516-3180.2014.8581009

**Published:** 2015-03-17

**Authors:** Miriam Raquel Diniz Zanetti, Carla Dellabarba Petricelli, Sandra Maria Alexandre, Aline Paschoal, Edward Araujo, Mary Uchiyama Nakamura

**Affiliations:** I PhD. Voluntary Physiotherapist, Pelvic Floor Unit, Department of Obstetrics, Universidade Federal de São Paulo (Unifesp), São Paulo, Brazil.; II MSc. Voluntary Physiotherapist, Pelvic Floor Unit, Department of Obstetrics, Universidade Federal de São Paulo (Unifesp), São Paulo, Brazil.; III PhD. Adjunct Professor, Pelvic Floor Unit, Department of Obstetrics, Universidade Federal de São Paulo (Unifesp), São Paulo, Brazil.; IV BSc. Postgraduate Student, Pelvic Floor Unit, Department of Obstetrics, Universidade Federal de São Paulo (Unifesp), São Paulo, Brazil.; V PhD. Associate Professor, Pelvic Floor Unit, Department of Obstetrics, Universidade Federal de São Paulo (Unifesp), São Paulo, Brazil.

**Keywords:** Physical therapy modalities, Pelvic floor, Perineum, Labor stage, first, Parturition

## Abstract

**CONTEXT AND OBJECTIVE:**

Several risk factors are involved in perineal lacerations during vaginal delivery. However, little is known about the influence of perineal distensibility as a protective factor. The aim here was to determine a cutoff value for pelvic floor distensibility measured using the Epi-no balloon, which could be used as a predictive factor for perineal integrity in vaginal delivery.

**DESIGN AND SETTING:**

Prospective observational single cohort study conducted in a maternity hospital.

**METHODS:**

A convenience sample of 227 consecutive at-term parturients was used. All women had a single fetus in the vertex presentation, with up to 9.0 cm of dilation. The maximum dilation of the Epi-no balloon was measured using a tape measure after it had been inflated inside the vagina up to the parturients’ maximum tolerance. The receiver operating characteristic (ROC) curve was used to obtain the Epi-no circumference measurement with best sensitivity and specificity.

**RESULTS:**

Among the 161 patients who were included in the study, 50.9% underwent episiotomy, 21.8% presented lacerations and 27.3% retained an intact perineum. Age > 25.9 years; number of pregnancies > 3.4; number of deliveries > 2.2 and circumference measured by Epi-no > 21.4 cm were all directly correlated with an intact perineum. Circumference measurements using the Epi-no balloon that were greater than 20.8 cm showed sensitivity and specificity of 70.5% and 66.7% (area under curve = 0.713), respectively, as a predictive factor for an intact perineum in vaginal delivery.

**CONCLUSION:**

Circumferences greater than 20.8 cm achieved using the Epi-no balloon are a predictive factor for perineal integrity in parturients.

## RESUMO

**CONTEXTO E OBJETIVO:**

Diversos fatores de risco estão envolvidos nas lacerações do períneo durante o parto vaginal, contudo, pouco se sabe sobre a influência da extensibilidade perineal como um fator protetor. O objetivo foi avaliar o ponto de corte da extensibilidade do assoalho pélvico medido pelo balão Epi-no, o qual poderia ser usado como fator preditor de integridade perineal no parto vaginal.

**TIPO DE ESTUDO E LOCAL:**

Estudo prospectivo observacional de coorte única conduzido em maternidade.

**MÉTODOS:**

Uma amostra de conveniência de 277 parturientes consecutivas no termo foi utilizada. Todas as mulheres tinham feto único com apresentação cefálica fletida, com até 9,0 cm de dilatação. A máxima dilatação do balão Epi-no foi medida com fita métrica após a sua insuflação dentro da vagina até a tolerância máxima da parturiente. Uma curva característica de operação do receptor (ROC) foi utilizada para obter a medida da circunferência com a melhor sensibilidade e especificidade.

**RESULTADOS:**

Dentre as 161 pacientes que foram incluídas no estudo, 50,9% sofreram episiotomia, 21,8% lacerações e 27,3% tiveram o períneo intacto. Idade > 25,9 anos; número de gestações > 3,4; número de partos > 2,2; e medida do perímetro do Epi-no > 21,4 cm foram todos diretamente correlacionados com períneo intacto. Os valores do perímetro com o balão Epi-no que estavam acima de 20,8 cm mostraram sensibilidade e especificidade de 70,5% e 66,7% (área sob a curva = 0,713), respectivamente, como fator preditor de períneo intacto no parto vaginal.

**CONCLUSÃO:**

Circunferência medida pelo balão Epi-no maior que 20,8 cm é fator preditor de integridade perineal em parturientes.

## INTRODUCTION

The pelvic floor muscles are a complex involving two layers of muscles. One layer involving the levator ani and puborectalis muscles is deeper and the other is more superficial and involves the perineum.^1^

Vaginal delivery has been considered to be an important predictive factor for pelvic floor dysfunction, including urinary or fecal incontinence, genital prolapse and levator trauma.^2^ This is due to the extensive stretching of the pelvic floor during delivery. Cesarean section reduces the risk of pelvic floor trauma but is not entirely protective.^3^

It has been proven that vaginal delivery increases the levator hiatal dimensions, especially after an avulsion injury.^4^ In a prospective cohort study on 39 women who delivered vaginally, three-dimensional translabial ultrasound was performed during the postpartum period and was repeated two and six months after delivery. Levator avulsion occurred in 39%, and vaginal delivery was correlated with higher maternal age, operative delivery and worsened stress incontinence postpartum.^5^ In another study, levator hiatal area > 25 cm in the Valsalva maneuver, measured by three-dimensional ultrasound, was defined as abnormal distensibility or “ballooning” of the levator hiatus.^6^

The most severe obstetric perineal lesions occur when the soft tissue, muscle, fascia, adipose tissue, skin and mucosa are not sufficiently extensible to permit fetal passage. However, these soft perineal tissues can distend, and the extent of the distension varies both between parturients and between pregnancies within an individual. Moreover, this distension can be reduced or increased during the course of the pregnancy by promoting shrinkage or stretching of the soft perineal tissues, respectively, using physiotherapeutic methods.^7^

Some risk factors for perineal trauma during vaginal delivery have already been established, and these include advanced maternal age, “Caucasian and Asian” races, high maternal body mass index, operative vaginal deliveries, a prolonged expulsive period and high birth weight of the newborn.^8^^,^^9^^,^^10^ However, there is a lack of studies on the importance of pelvic floor distensibility and its relationship with birth trauma. Distensibility of the perineum is very important during the second stage of labor, for preventing birth trauma, because of the high pressure imposed by the fetus head on the muscles of the pelvic floor.^11^

The Epi-no Delphine Plusvaginal dilator (Starnberg Medical, Tecsana GmbH, Munich, Germany) consists of an inflatable silicone balloon connected to a manometer via a rubber tube.^12^ Recently, Kubotani et al.^13^ compared perineal distensibility using Epi-no in 23 singleton and 20 twin pregnancies. There was no difference in perineal distensibility between the two groups, but there was a positive correlation between perineal distensibility and abdominal circumference in twin pregnancies.

## OBJECTIVE

Because of the absence of an instrument for objectively and quantitatively assessing the maximum degree of pelvic floor distensibility, we decided to use the Epi-no device as a method for measuring this biomechanical property. Thus, the aim of this study was to determine a cutoff value, in centimeters, for pelvic floor distensibility measured using the Epi-no balloon, which could be used as a predictive factor for muscle integrity in vaginal delivery.

## METHODS

A prospective observational single cohort study was conducted at the Amador Aguiar Maternity Hospital (HMMAA), in Osasco, state of São Paulo, Brazil, between January and December 2009. The project was evaluated and approved by the Research Ethics Committee of Universidade Federal de São Paulo (Unifesp), under registration number 1283/08, and by the National Research Ethics Committee, under report number 676. HMMAA is the largest public maternity hospital in Osasco and provides care for low-risk pregnancies (70%) and high-risk pregnancies (30%), at a rate of 600 deliveries/month.

The study included 227 consecutive at-term single births in the cephalic presentation with up to 9.0 centimeters of dilatation and at a maximum station of zero, based on the American College of Obstetrics and Gynecologists classification of fetal head station assessments.^14^ We included both primiparous and multiparous parturients. Only collaborative parturients who wished to undergo the examination, who had not received anesthesia (e.g. rachidian, peridural or combined block) and whose fetus showed good vitality at the time of the assessment were included.

Patients firstly read and signed the informed consent form. If the patient was still a teenager, her mother needed to provide consent and sign for her. The participants then underwent pelvic floor distensibility assessment (comprising pelvic floor and perineum), which was measured as the circumference in centimeters of the inflated balloon of the Epi-no device (Starnberg Medical, Tecsana GmbH, Munich, Germany). This was done upon admission to the delivery room. The Epi-no circumference measurements were made by a single examiner (MRDZ), who had had four years of experience of using the Epi-no balloon for perineal muscle training during pregnancy. To reduce the bias of individual tolerance, all parturients received information regarding the safety of this device through the assurance that its use does not increase the risk of vaginal infection.^15^

For the test, the parturients were placed in the dorsal decubitus position with flexed and abducted lower limbs (from 30° to 45°) and with their feet supported on the bed. They were asked not to contract their gluteal, perineal or adductor muscles. The balloon was covered with a condom and, after application of a gel lubricant, was introduced into the vagina until only two centimeters were visible outside the vaginal introitus. This was the assurance that the balloon had reached not only the superficial layer of the pelvic floor (perineum) but also the deepest layer (including the levator ani muscle). The balloon was then gradually inflated until the tolerable limit, which was subjectively determined by the patient, was reached. All of the patient assessments were performed by the same examiner. Next, the balloon was slowly withdrawn while still fully inflated, the condom was removed and the largest circumference of the balloon was measured using a measuring tape.

The sample size was estimated such that sufficient precision would be attained, i.e. a 95% confidence interval (CI) of width = 0.20, if the observed area under receiver operating characteristic (ROC) curve was greater than 0.60.^16^ For an the area under the ROC curve of 0.713, we would need to assess 160 subjects to have a 95% CI width ≤ 0.20.

The perineal trauma was classified based on third-degree laceration (when the extent of the lesion included the external anal sphincter totally or partially) and fourth-degree laceration (when the rectal mucosa was involved).^17^ The diagnosis of perineal trauma was made both by doctors and by the midwifes who assisted the labor, but the repairs were made only by doctors.

Statistical analysis was performed using Statistical Package for the Social Sciences (SPSS) v.14 (SPSS Inc., Chicago, IL, USA) and Minitab v.13 (Minitab Inc., State College, PA, USA). The sample size used for our study provided a power of 82.7%. First of all, descriptive statistics were produced on all the variables studied (age, number of gestations and deliveries, body mass index, pelvic floor muscle extensibility, newborn weight and newborn cephalic circumference). Next, univariate analysis was applied to determine which variables influenced perineal outcomes. Student’s t test for analysis of continuous variables and the Mann-Whitney test were used when the data were not normally distributed. After that, multivariate logistic regression was used, taking into consideration all the significant variables of the univariate analysis at a significance level of 20%. Adjusted multivariate logistic regression was performed by means of a backward process. Appropriate odds ratios (OR) with 95% CI were calculated. Probability values < 0.05 were regarded as statistically significant.

## RESULTS

Initially, we assessed 227 parturients, of whom 117 were nulliparous and 110 were multiparous. White, mixed and black skin color corresponded to 45.8%, 44.9% and 8.8%, respectively. Following delivery, 66 patients (29.1% of the cohort) were excluded from the analysis: 57 (25.1%) because their delivery was via cesarean section, eight (3.5%) because they did not provide sufficient medical data and one (0.44%) because the patient left the hospital against medical advice. There was no use of forceps or vacuum extractor device for assisting in any parturient’s delivery.

The patients were not followed up after delivery, because the hospital where this study was conducted is a public hospital that only provides delivery care, while puerperium follow-up is provided at several primary healthcare units in the metropolitan region of São Paulo. Hence, proper follow-up for perineal trauma cases was impossible.

The 161 remaining parturients averaged 23.6 ± 5.1 years of age with an average body mass index of 27.6 ± 4.3 kg/m^2^. The patients had an average Epi-no balloon maximum circumference of 19.9 ± 2.7 cm and gave birth to newborns that weighed 3,168 ± 428 g with a head circumference of 34.1 ± 1.5 cm.

With regard to the perineal outcomes of the 161 patients who were included, 50.9% (n = 82 patients) received right mediolateral episiotomy, 21.8% (n = 35) suffered laceration and 27.3% (n = 44) maintained an intact perineum. The perineal outcomes were then analyzed based on variables including age, number of pregnancies, parity, body mass index, Epi-no balloon circumference, newborn weight and newborn head circumference. These parameters are presented in Table 1.

**Table 1.**
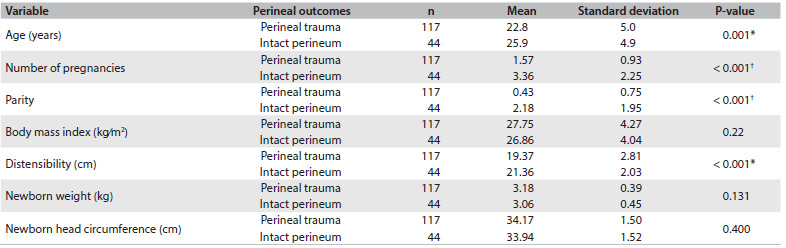
Univariate analysis from predictive factors for perineal integrity after vaginal delivery

^*^Student-t test; ^†^Mann-Whitney test.

The results from adjusted multivariate logistic regression using the backward process are presented in Table 2. This shows that greater parity, higher distensibility (Epi-no balloon values) and lower newborn weight were predictive factors for perineal integrity.

**Table 2.**
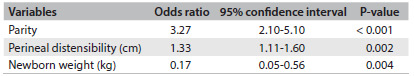
Final results from multivariate logistic regression using backward process

**Figure 1.**
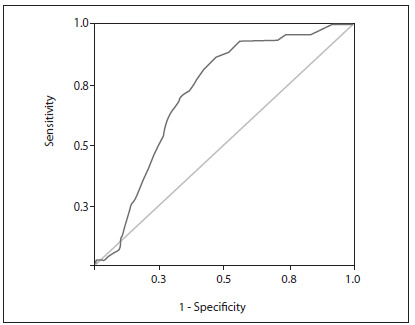
Receiver operating characteristic (ROC) curve for perineal integrity assessment using the *Epi-no* circumference measurement. Area under curve = 0.713; sensitivity of 70.5% and specificity of 66.7%.

The ROC curve was constructed, and this demonstrated that an Epi-no circumference measurement of 20.8 cm was the best cutoff for perineal integrity after vaginal delivery (area under curve = 0.713; sensitivity of 70.5% and specificity of 66.7%) (Figure 1).

## DISCUSSION

According to Astrand and Rodahl,^18^ muscle fibers have biomechanical properties such as excitability, contractility, distensibility and elasticity. Distensibility and elasticity differ because the former property indicates the extent to which a fiber can distend during a stretch stimulus, and the latter indicates how well the fiber can return to its original length following the stretch stimulus.

To the best of our knowledge, no previous study has objectively investigated the maximum distensibility of the pelvic floor muscles. Shek and Dietz^19^ studied the influence of levator ani distensibility on occurrences of levator avulsion after vaginal delivery. They concluded that the levator avulsion could not be predicted antenatally, but they measured the pelvic floor distensibility using transperineal ultrasound when the women were doing a Valsalva maneuver. It is possible that, during this maneuver, the levator muscle does not achieve its maximum distensibility, because for this to occur, it would have to be stretched passively, i.e. the muscle would need to be relaxed while a movement elongating it and separating its origin from its insertion was being performed.^20^ However, our study did not have the objective of assessing levator avulsion.

In the present study, an inflatable balloon was introduced into the vagina and was inflated to produce substantial distension of the pelvic floor muscles. A measure of muscle distension can then be obtained by measuring the circumference of the fully inflated balloon. Although the device was not originally designed for this purpose, this adaptation was necessary because no alternative method for measuring perineal distensibility is currently available.

One of the most frequent complaints from patients regarding vaginal birth is the fear of a perineal lesion (as occurs with episiotomy or laceration, for example) that could lead to sexual dysfunction after delivery.^21^ In some Latin American countries, including Brazil, the incidence of cesarean sections is as high as 80% in private care and leads to persistent concern within the healthcare system.^22^ Introduction of a test that could predict the likelihood that a lesion would not occur might allow the expectant mother to be more comfortable and “secure” in opting for vaginal delivery. In this regard, predictive factors for pelvic floor lesions during vaginal birth should be studied further. Although some factors predictive of lesions have been identified, such as a prolonged expulsion period, a macrosomic fetus, advanced maternal age, ethnicity and high body mass index, pelvic floor distensibility has received little attention.

Two previous studies investigated the use of the Epi-no balloon trainer to prepare the perineum for vaginal delivery and to reduce levator trauma. In the first of these, Ruckhäberle et al.^15^ conducted a prospective, randomized study using Epi-no during pregnancy for perineal preparation (to increase muscle extensibility) prior to birth. A total of 135 primigravidae participated in the study and used the device for at least 15 minutes per day from the 37^th^ gestational week onwards, for an average of 15 consecutive days; a control group of 135 primigravidae did not undergo any perineal preparation. After training, the study group had a mean circumference of 24.3 ± 4.4 cm and showed a tendency towards increased likelihood of having an intact perineum, in comparison with the control group (P = 0.05).

However, there are some possible caveats to the study by Ruckhäberle et al.^15^ First, the women were instructed to perform the stretching exercise with the Epi-no device at home without any previous supervised training. Because using the Epi-no is not straightforward, this could lead to a bias in its application and in the subsequent results. In addition, the pregnant women themselves were instructed to measure the maximum balloon circumference, which could also introduce a bias in the results. Such considerations might explain the primary difference between our results and theirs, in which they reported larger Epi-no circumference measurements. It is important to note that all of our measurements were conducted by the same examiner (MRDZ), which might have produced more reproducible data.

In the second of these investigations on the Epi-no trainer, Shek et al.^23^ conducted a randomized controlled trial to assess if the pelvic trainer could reduce levator trauma. The authors selected 200 nulliparous women with singleton pregnancies, and these patients were divided into intervention and control groups. These patients were examined by means of three-dimensional translabial ultrasound at 35-37 weeks and three months after delivery. The patients in the intervention group were instructed to use the Epi no device from the 37^th^ week onwards. A total of 156 women returned for the follow-up examination, of whom 78 had had vaginal deliveries. The risk of avulsion was halved in the intervention group (6% versus 13%; P = 0.19). The analysis on the treatment received revealed that the intervention group presented nonsignificant 42% and 30% reductions in levator avulsion and microtrauma, respectively (P ≥ 0.22). The authors concluded that the Epi no balloon did not reduce the incidence of levator trauma.

One limitation of the present study is that it was difficult for a single examiner to operate the device without assistance. Thus, two examiners were required (AP and CDP), in which the principal researcher introduced the balloon to the correct depth and held it in place while the second examiner inflated the balloon. In addition, it would be also very important to evaluate the patients after delivery, using three-dimensional ultrasound to investigate occurrences of levator avulsion, as done by Dietz and Shek.^24^

In our study, we observed no bleeding, which is consistent with the report by Ruckhäberle et al.,^15^ or any other serious complaint. This suggests that it is safe to use this equipment. One previous study reported on a patient who used the Epi-no device and suffered venous air embolism.^25^ In this case, the patient’s husband helped her to inflate the device, and after ten minutes of inflation, the patient began to complain of vaginal pain and dizziness, after which the device was immediately removed. Following a convulsive period, she became unresponsive and was taken to the emergency service, where a cesarean section was performed, followed by care in the surgical intensive care unit. After two months, she no longer exhibited any neurological sequelae but was counseled regarding the risk of uterine rupture in future pregnancies. The authors of the report hypothesized that the Epi-no device had had an unobserved leak that led to the severe complications. To avoid this potential complication, in the present study we covered the balloon with a condom, which prevented the entry of air during inflation of the balloon. Moreover, although the device has been described as simple to use, we believe that use by an unsupervised non-professional can be harmful. However, new studies about the safety of the Epi-no balloon when it is used by healthcare professionals or people without previous training should be conducted to prove the real degree of safety of the Epi-no device.

From a clinical perspective, when a pregnant woman presents a rigid perineum, she could perform local stretching, for example by means of perineal massage and/or use of an Epi-no device, to achieve adequate perineal distensibility.

Some authors have reported that the levator ani muscle can distend during fetal head descent, during vaginal delivery. Lien et al.^26^ performed computer simulations on vaginal childbirth and demonstrated that the pubovisceral portion of the levator ani muscle is subject to a stretch ratio of more than 3:1. Similar results were reported by Hoyter et al.,^27^ who used magnetic resonance imaging of a nulligravid pelvic floor to create a simulation model and found that the puborectalis muscle can reach a stretch ratio of 3.5:1 during fetal head descent. Although these studies are important for providing indirect knowledge regarding the mechanism of muscle stretching during a vaginal delivery, such simulations cannot consider the mechanical properties of the pelvic floor with regard to the important biomechanical changes that occur during pregnancy and delivery.^27^

In the present study, we evaluated the pelvic floor during maximum stretching during parturition, at which the biomechanical distensibility was at its maximum level. However, the question still remains as to whether these muscles might suffer a more intrinsic, nonvisible form of perineal lesion such as levator avulsion. Thus, this study presented a new method for assessing distensibility, which will allow future researchers to understand the importance of distensibility in conferring protection to the pelvic floor during childbirth.

We believe that the main bias of our study was the inflation of the Epi-no balloon up to the tolerable limit, which was subjectively determined by the patient. However, all patients received information regarding the safety of the Epi-no balloon before using it.

## CONCLUSION

In summary, a circumference achieved by the Epi-no balloon that was larger than 20.8 cm was a predictive factor for perineal integrity in these parturients. New studies with large population samples are necessary to prove our results.
